# Detection, Localization and Quantification of Impact Events on a Stiffened Composite Panel with Embedded Fiber Bragg Grating Sensor Networks

**DOI:** 10.3390/s17040743

**Published:** 2017-04-01

**Authors:** Alfredo Lamberti, Geert Luyckx, Wim Van Paepegem, Ali Rezayat, Steve Vanlanduit

**Affiliations:** 1 Department of Materials, Textiles and Chemical Engineering (MaTCh), Ghent University, Technologiepark 903, 9052 Zwijnaarde, Belgium; geert.luyckx@ugent.be (G.L.); wim.vanpaepegem@ugent.be (W.V.P.); 2 Department of Mechanical Engineering, Vrije Universiteit Brussel (VUB), Pleinlaan 2, 1050 Elsene, Belgium; arezayat@vub.ac.be; 3 Faculty of Applied Engineering, University of Antwerp, Campus Hoboken Salesianenlaan 90, 2660 Antwerp, Belgium; Steve.Vanlanduit@uantwerpen.be

**Keywords:** fiber Bragg grating, optical sensing, composite materials, impact identification, inverse methods

## Abstract

Nowadays, it is possible to manufacture smart composite materials with embedded fiber optic sensors. These sensors can be exploited during the composites’ operating life to identify occurring damages such as delaminations. For composite materials adopted in the aviation and wind energy sector, delaminations are most often caused by impacts with external objects. The detection, localization and quantification of such impacts are therefore crucial for the prevention of catastrophic events. In this paper, we demonstrate the feasibility to perform impact identification in smart composite structures with embedded fiber optic sensors. For our analyses, we manufactured a carbon fiber reinforced plate in which we embedded a distributed network of fiber Bragg grating (FBG) sensors. We impacted the plate with a modal hammer and we identified the impacts by processing the FBG data with an improved fast phase correlation (FPC) algorithm in combination with a variable selective least squares (VS-LS) inverse solver approach. A total of 164 impacts distributed on 41 possible impact locations were analyzed. We compared our methodology with the traditional P-Inv based approach. In terms of impact localization, our methodology performed better in 70.7% of the cases. An improvement on the impact time domain reconstruction was achieved in 95.1% of the cases.

## 1. Introduction

Composite materials constitute a better alternative to classical metals in many application fields. Composites are, in fact, characterized by higher specific mechanical properties (i.e., mechanical properties per unit weight) as well as better corrosion and fatigue resistance capabilities. Compared to metals, though, composites can show some additional damage mechanisms, such as delaminations, matrix cracks, matrix-fiber debonding, etc. Much of this damage occurs internally and is often undetectable by inspecting the composite external surface. Such damages can propagate unseen under the action of external operating loads, and they can eventually lead to catastrophic events. The identification of the causes that can lead to the onset of a structural damage is vital. For composites operating in the aerospace and wind energy sectors, it is known that one of the major causes of damage is represented by impacts with external objects. The capability to detect, localize and quantify impact events can therefore help the damage detection and the composite structural health monitoring. Impact identification can be achieved by measuring the impact induced structural vibrations and by adopting advanced signal processing techniques. Several sensing devices are available on the market to measure vibrations, such as strain gauges, accelerometers and fiber optics. Optical fibers have been more and more used in the last years since they offer several advantages. Besides being immune to electromagnetic interference and resistant to corrosion, they are small in size and extremely light. Therefore, they can be surface mounted or even embedded in composite materials without causing excessive structural modifications. Furthermore, some types of fiber optic sensors, such as fiber Bragg grating (FBG) [1,2], allow for spatially multiplexing up to dozens or even hundreds of sensors by using only one optical fiber cable. Distributed surface mounted FBGs have already been reported in literature for impact identification problems [3,4,5,6]. In 2010, Coelho et al. [4] identified impacts occurring on a carbon fiber reinforced (CFR) composite wing with multiplexed surface mounted FBG sensors. The localization method they proposed is based on the maximum strain amplitude measured by FBG sensors during impact and the relative placement of all sensors. In 2012, Jang et al. [3] proposed a neural network method for impact localization using the impact-induced acoustic waves acquired by multiplexed FBG sensors. This methodology relies on a high speed (100 kHz) FBG interrogation device that allows for retrieving reliable time of arrival (TOA). TOA techniques are, however, difficult to apply on complex structures and present high noise sensitivity, being therefore unsuitable for the identification of colored-band excitation. Moreover, in order to achieve an accurate impact localization, TOA methods require knowing the wavelength propagation speeds in the structure as well as the exact sensor placement. Distributed surface mounted FBGs have recently been used by the authors to identify impacts occurring on flat carbon fiber reinforced plates [6]. In particular, we proposed a model-based identification approach that performs the impact identification by exploiting the FBG strain signals together with the strain frequency response functions (SFRF). In such methodology, the knowledge of the FBG sensors’ location is not required.

In many cases, surface mounted FBGs are not ideal. Surface mounted FBGs can, for instance, be damaged in the case of impacts. Embedded FBGs can overcome this issue and provide more meaningful data by measuring internal strains. The main drawback of using embedded FBGs is associated with the chance of having severe optical signal distortion [7,8,9,10], which can negatively affect the reliability of the measurements. Nevertheless, many studies have shown that, by embedding the optical fiber lines parallel to the direction of the composite fiber reinforcements [9,10] and by adopting dedicated signal processing [11,12,13,14], the optical signal distortion can be managed and overcome. In this paper, we demonstrate the possibility to fully identify impacts on a complex real-life composite structure with embedded FBG sensors. For such a purpose, we decided to used a model-based methodology operating in the frequency domain. Model-based techniques, in fact, can deal with structures having a complex material composition as well as a complex geometry. This is often the case for composite components. For our investigation, we used a CFR composite plate reinforced with CFR omega shaped stiffeners. During the manufacturing of such a stiffened plate, we embedded three optical fiber (OF) lines with a total of 18 multiplexed FBGs. The plate was mounted in an ad hoc designed support frame and impacted with a modal hammer. We considered 41 possible impact locations and, for each one of them, we repeated the impact test four times in order to retrieve some statistical indicators. Overall, 164 impacts were performed. For each impact, we analyzed the data obtained via the embedded FBGs with a two-step methodology. In the first step, we demodulated the FBGs optical spectra using an improved version of the fast phase correlation (FPC) algorithm proposed by Lamberti [11]. In such a way, we obtained accurate measurements of the impact induced internal dynamic strains. In the second step, we processed the dynamic strains with a variable selective least squares (VS-LS) inverse solver approach [6]. As output of this two-step methodology, we obtained the estimated impact location as well as the reconstruction of the impact force time histories. Comparing these estimations with the known impact locations and with the measurements of the modal hammer, we were able to retrieve some statistical indicators of the accuracy of our approach. From the point of view of impact localization, our estimation was correct (i.e., without any uncertainty) in 78% of the cases. The reconstruction of the impacts was in very good agreement with the measured forces even in the cases where the localization error was high. This was due to the nature of the VS-LS algorithm, which solves the inverse problem related to impact reconstruction independently from the localization. The experimental results also showed how the proposed methodology outperforms the standard pseudo-inverse (P-Inv) based approach.

The article is structured as follows. Section 2 recalls the theoretical aspects used throughout this work. It first presents the FBG principles and the improved FPC algorithm (Section 2.2) and then discusses the VS-LS method. Section 3 is dedicated to the manufacturing of the stiffened CFR panel used for the analysis. The experimental setup and procedure are described in Section 4. This section also discusses the experimental results and provides the statistical metrics regarding the impact localization and reconstruction accuracy. Finally, Section 5 summarizes the findings and contains the conclusive remarks.

## 2. Theoretical Aspects

This section starts by recalling the theory of FBG sensors and by introducing the mathematical formulation of the improved FPC algorithm adopted for the processing of the FBG signals. Successively, it recalls the principle of the VS-LS inverse solver exploited for the impact localization and time reconstruction.

### 2.1. FBG Principles and Spectra Demodulation

**Figure 1:**
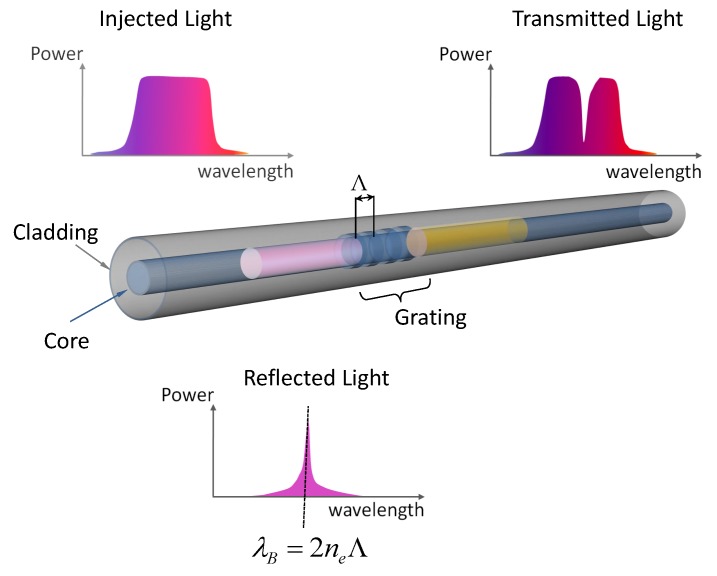
Schematic representation of the fiber Bragg grating (FBG) working principle.

An FBG sensor is an optical fiber in which a grating is created inside the fiber core region by exposing the optical fiber to UV light. The grating is a modulation of the material refractive index and acts as a wavelength selective filter. When broadband light is sent into the fiber (see Figure 1), the grating transmits all the wavelengths except for one, which is reflected back. Such a wavelength is called the Bragg wavelength and is expressed as $\lambda_{B}=2n_{e}\Lambda$, where $n_{e}$ indicates the effective refractive index of the material and Λ is the grating pitch. When the fiber is strained, the Bragg wavelength $\lambda_{B}$ shifts in the wavelength domain: a tension load produces positive $\Delta \lambda_{B}$ (the $\lambda_{B}$ shifts towards higher wavelengths) while a compression force induces negative $\Delta \lambda_{B}$ (the $\lambda_{B}$ shifts towards lower wavelengths).

The tracking of the Bragg wavelength shifts allows for measuring the applied strain level. For silica fibers operating in the 1550 nm wavelength range, the strain sensitivity is approximately 1.24 pm/μϵ. There are several algorithms available in literature to compute the Bragg shift [15]. Some of them simply track the position of the point of maximum reflected power. These methods are, however, not reliable when the Bragg peak distorts. Bragg peak distortions such as peak splitting and broadening can occur when FBGs are embedded in composite materials. The reasons for this are associated to the complicated non uniform strain distribution that may act on the fiber grating. One precaution that can be adopted to reduce such a phenomenon is related to the embedding of the optical fiber line parallel to the direction of the composite reinforcement fibers. In some cases, though, this is not possible, especially when the geometry of the composite component is complex or when a cross ply stacking sequence is needed. In this case, there are some other algorithms that can deal with the distorted FBG signal. One of these is the Fast Phase Correlation (FPC) algorithm [11]. The advanced capability of such an algorithm has been demonstrated in several publications. In this paper, we will use an improved version of the FPC algorithm. In such a version, the $\Delta \lambda_{B}$ is computed as follows:

(1) $$\Delta \lambda_{B}=\underset{2\leq k\leq M}{\mathrm{median}}\;\left(\frac{R^{\prime }\left(k\right)}{R(k)}\frac{N\;\delta \lambda }{2\pi (k-1)}\right),$$

where $R^{\prime }\left(k\right)$ is the Fourier transform of the reference initial FBG spectrum, R(k) is the Fourier transform of the FBG shifted spectrum (after the load is applied), *N* is the number of sampling points of the FBG spectrum, δλ represents the FBG spectral resolution, *k* indicates the Fourier spectral line number and *M* is the maximum number of spectral lines used for the computation. Compared to the original FPC, this version calculates the phase shift between two FBG spectra by taking the ratios of $R^{\prime }\left(k\right)$ with R(k) rather than subtracting the phases obtained individually from $R^{\prime }\left(k\right)$ and R(k). In such a way, the risk of phase wrapping errors is eliminated.

### 2.2. The Variable Selective Least Squares (VS-LS) Inverse Solver

The VS-LS solver has been recently proposed by the authors [6]. Although its performance has been already tested on demodulated strain signals obtained by surface mounted FBGs, it has never been applied to signals retrieved by embedded FBGs. The VS-LS is a model based approach operating in the frequency domain. It is structured to find a solution to the following set of equations:

(2) $$E^{f}=H^{\epsilon f}F^{f},\;f\in \left\{f_{1},\ldots ,f_{N_{f}}\right\},$$

where E is the n×1 vector of measured internal strains, $H^{\epsilon }$ is the n×ℓ matrix of strain frequency response functions with *ℓ* the number of possible impact locations, and *F* is the ℓ×1 vector of occurred impacts. The superscript *f* indicates the frequency line while $N_{f}$ is the number of frequency lines in the studied range. The $H^{\epsilon f}$ is, in fact, the frequency domain model of the structure under test and needs to be preliminary assembled in order to make the VS-LS estimate the impacts *F* from the measured strain E. The $H^{\epsilon f}$ matrix can be determined either numerically (using a finite element model of the test structure) or experimentally, for instance by means of a roving hammer test [16]. In this work, $H^{\epsilon f}$ is experimentally constructed via a preliminary roving haummer test. In particular, the $H^{\epsilon f}$ is here constructed using a so-called $H_{1}$ method (i.e., assuming noise only on the output). For each possible impact location *ℓ*, three impacts are performed and the correspondent strain transfer functions are computed as $H_{1}=\bar{\text{ XPS }}/\bar{\text{ APS }}$, where $\bar{\text{ XPS }}$ indicates the averaged cross-power spectrum between the input (measured forces) and output (measured strains) signals and $\bar{\text{ APS }}$ is the averaged auto-power spectrum of the inputs. A frequency domain system identification is then applied to the measured strain frequency responses in order to build the structure-model $H^{\epsilon f}$. This step involves the calculation of the system modal parameters (resonance frequency, damping ratios, mode shapes and participation factors) and is performed using the state-of-the-art polyreference least-square complex frequency-domain (PolyMAX) [17] estimator. The PolyMAX estimator eases the selection of the system modal parameters and guarantees a high quality system model, which is indispensable for obtaining reliable results in the inverse procedure involved in this impact identification problem. It is worth noticing that, since the number *n* of embedded FBGs (i.e., measurement points) is lower than the number of possible impact locations *ℓ*, the $H^{\epsilon f}$ is a rectangular matrix, indicating that the problem is under-determined. Once $H^{\epsilon f}$ is available, an impact occurring in one of the possible *k* locations is localized and reconstructed as follows:

(3) $$\begin{array}{cc} \hat{\ell } & ={\mathrm{argmin}}_{\ell }\left\{\sum_{f=f_{1}}^{f_{N}}{∥{H^{\epsilon }}_{j}^{f}{\tilde{F}}_{j}^{f}-E^{f}∥}_{2}^{2}\right\},\;j=1:\ell , \end{array}$$

(4) $$\begin{array}{cc} \hat{F^{f}} & ={\left[{H^{\epsilon }}_{\hat{\ell }}^{f}\right]}^{†}E^{f}. \end{array}$$

In Equation (3), $\hat{\ell }$ is the estimated impact location resulting from the minimization of the cost function $CF=\sum_{f=f_{1}}^{f_{N}}{∥{H^{\epsilon }}_{j}^{f}{\tilde{F}}_{j}^{f}-E^{f}∥}_{2}^{2}$. ${\tilde{F}}_{j}^{f}$ is the impact force estimated at the *j*-th impact location for each frequency line *f*; it is obtained by multiplying the P-Inv of $H_{\hat{k}}^{\epsilon f}$ with the vector $E^{f}$. Once $\hat{k}$ is known, Equation (4) computes the impact force in the frequency domain by multiplying the P-Inv of the strain frequency response matrix evaluated at $\hat{\ell }$ for the vector of the measured internal strain. The symbol † in Equation (4) indicates the P-Inv operator. The time domain reconstruction of the impact is eventually calculated by taking the inverse Fourier transform of Equation (4).

## 3. Material

**Figure 2:**
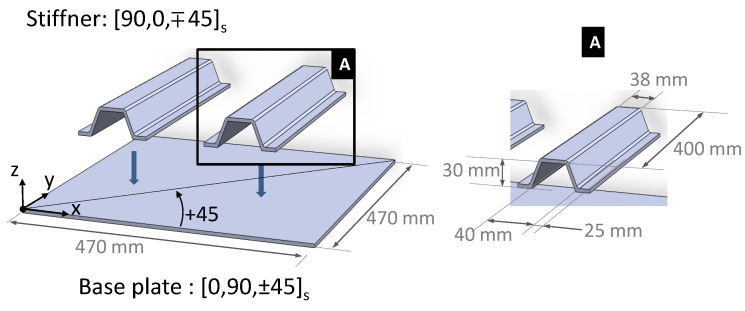
Schematic representation of the stiffened panel used for the analysis. The carbon reinforcements at 0° are aligned with the *x*-direction.

A schematic of the composite test structure used in this study is shown in Figure 2. The structure is constituted by two main sub-parts: a flat plate of 470 mm × 470 mm and two omega shape stiffeners whose dimensions are shown on the right side of Figure 2. Both the plate and the stiffeners were manufactured using M10/T300 carbon fiber reinforced pre-impregnated unidirectional layers. The mechanical properties of this material are reported in Table 1. In accordance with the Cartesian reference system shown in Figure 2, the stacking sequences adopted for the plate and the stiffeners were, respectively, [0/90/±45]_s_ and [0/90/∓45]_s_.

**Figure 3:**
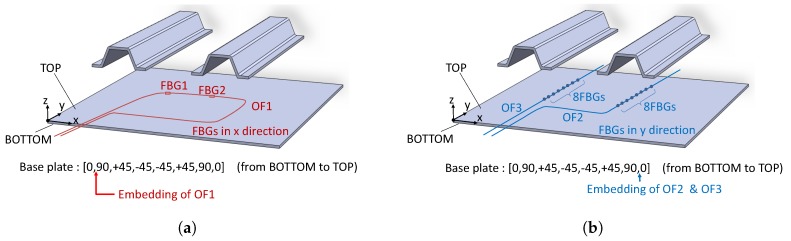
Placements of the three optical fiber (OF) lines inside the stiffened composite panel. OF1 (**a**) carries two gratings and is embedded between the 0/90 pre-preg layers at the bottom of the panel. OF2 and OF3 (**b**) carry eight gratings each and are embedded between the 90/0 pre-preg layers at the top side of the panel (closer to the stiffeners).

**Figure 4:**
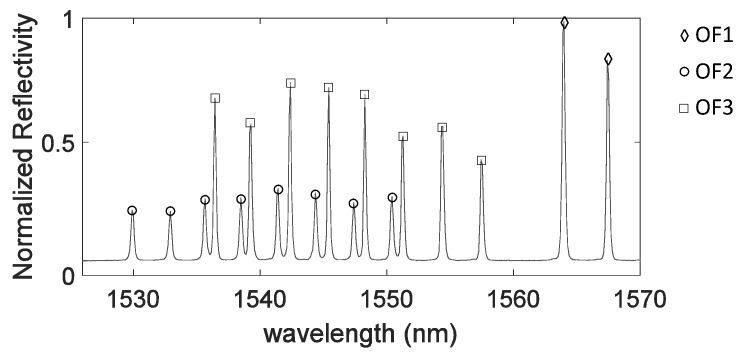
FBG spectrum after embedding and production. The peaks corresponding to the FBGs embedded in OF1, OF2 and OF3 are indicated, respectively, by ⟡, ∘, □.

During the placement of the CFR layers, three optical fiber (OF) lines were embedded (see Figure 3). OF1 was embedded between the 0/90 pre-preg layers at the bottom of the plate (i.e., on the plate side not attached to the stiffeners). It had two FBGs embedded, both with grating length of 8 mm and spaced 250 mm one from the other. OF2 and OF3 were embedded between the 90/0 pre-preg layers at the top side of the plate (i.e., on the plate side attached to the stiffeners). Both fibers were aligned in the *y*-direction of Figure 3b: OF2 was laid in the region below the right stiffener while OF3 was laid in the region below the left one. OF2 had eight FBGs, with grating length of 4 mm and grating separation of 20 mm. OF3 carried eight FBGs, with grating length of 4 mm and grating separation of 20 mm. Once the stacking sequence was prepared, the plate and the two stiffeners were cured in an oven for 8 h at 120 °C. In the final step, the stiffeners were glued to the plate using the commercially available adhesive FM 300 KO8 of CytecFM^®^. Figure 4 shows the Bragg wavelengths of the 18 FBGs at the end of the panel manufacturing process.

## 4. Experiments and Results

**Figure 5:**
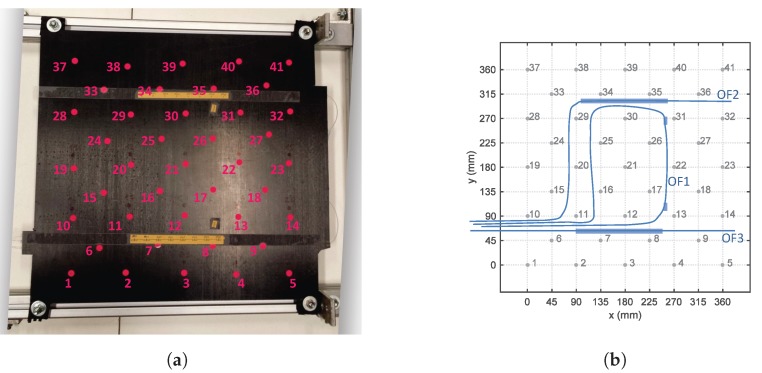
Experimental setup and impacts locations. (**a**) the 41 magenta grid points correspond to the possible impact locations while the yellow marks indicate the regions where the embedded FBGs are located. (**b**) impact grid with OF lines (light blue).

The CFR stiffened panel was mounted on an aluminum frame designed in order to clamp the panel at the four corners. Figure 5 shows the experimental setup and its schematic representation. In Figure 5a, the 41 possible impact locations are indicated by magenta points while the network of embedded FBGs is in yellow. Figure 5b is a schematic representation of the setup and provides the distance between the selected impact points. The points laying on the same horizontal (or vertical) line are 90 mm apart from each other. On the contrary, the points laying in diagonal directions are (approximately) 63.6 mm away from each other. In this paper, we refer to this last grid point separation as the “grid resolution”.

**Figure 6:**
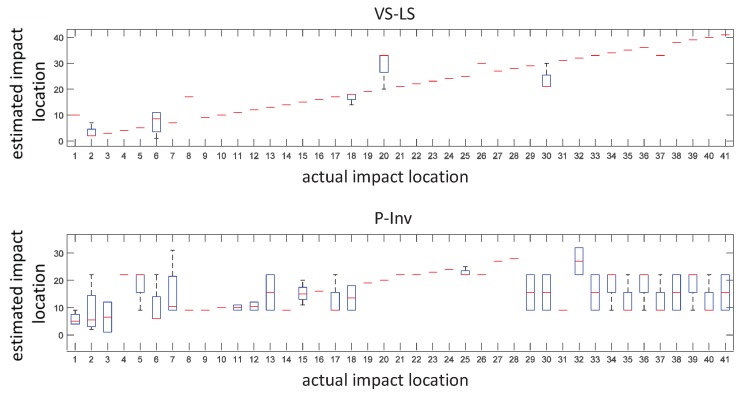
Estimated vs. actual impact locations. Comparison between (VS-LS) (**top**) and P-Inv (**bottom**) methods.

**Figure 7:**
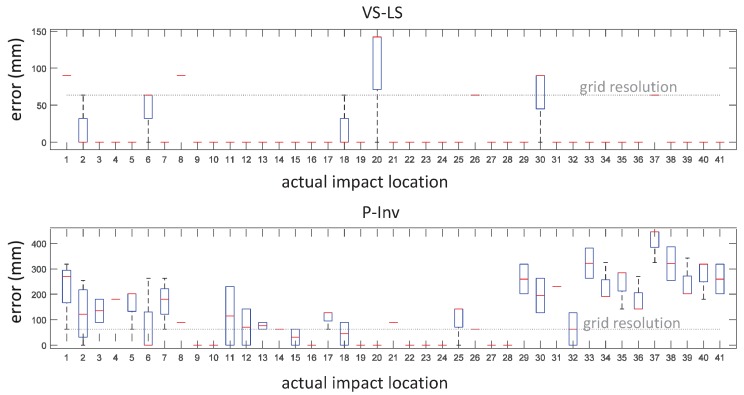
Localization error. Comparison between VS-LS (**top**) and P-Inv (**bottom**) methods.

Once the setup was ready, the plate was impacted using a modal hammer. For each of the selected impact points, four impacts were performed. During the impacts, the impact force and the internal strain were synchronously measured by using a National Instruments USB-6341 data acquisition card (National Instruments, Austin, TX, USA) [18] and a FBG scan 700 [19] interrogator (FBGS Technologies GmbH , Jena, Germany) (wavelength range 1525–1565 nm and resolution 78 pm) both driven by an in-house developed Matlab^®^ code (R2015b, MathWorks, Matick, MA, USA). A total of 164 impacts were performed. Successively, the acquired data were processed using the FPC algorithm and the VS-LS methodology described in Section 2. The estimated impact locations vs. the actual ones are reported in the box-and-whisker plot of Figure 6. Compared to the conventional P-Inv approach, our VS-LS method showed higher localization performances in 70.7% of the cases. The most evident improvement regarded the impact locations comprised in the range 30–41. In 78% of the cases (i.e., 32 locations out of 41), the VS-LS localized the impact with no uncertainty, meaning that, for each location, all four of impacts were exactly localized. The error on the impact localization is shown in the box-and-whisker plot of Figure 7. In this figure, the error is calculated as the Euclidean distance between the estimated and actual impact location. The VS-LS localization error was above the grid resolution (63.6 mm) only for four locations (1, 8, 20 and 30 in top of Figure 7).

The maximum localization error for the VS-LS was 164.3 mm and occurred at location 20 (Figure 7, top). For the P-Inv approach (Figure 7, bottom), the localization error was above the grid resolution limit in 70.7% of the cases, with the highest error of 445.5 mm occurring at location 37.

**Figure 8:**
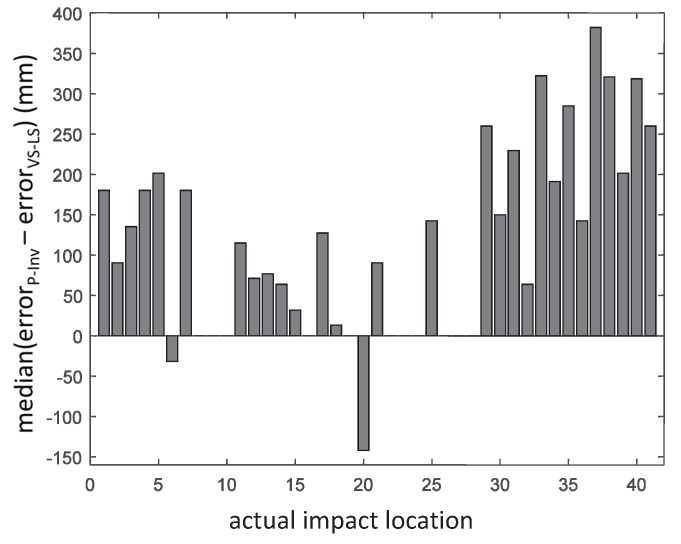
Median difference between P-Inv and VS-LS in terms of localization error.

**Figure 9:**
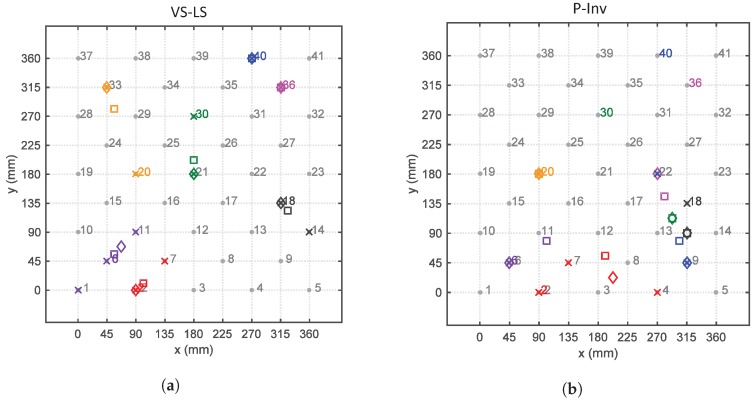
Schematic spatial representation of the impact location for location 2 (red), 6 (violet), 18 (black), 20 (orange), 30 (green), 36 (magenta) and 40 (blue). Comparison between VS-LS (**a**) and P-Inv (**b**): the × indicates each one of the four impacts; the □ indicates the mean; and the ⟡ indicates the median.

The median value of the difference between the localization errors of the P-Inv and VS-LS approaches is reported in Figure 8. The VS-LS produced a higher error than the P-Inv approach only for two locations (6 and 20) out of the 41 possible ones. In all of the other locations, the localization error of the VS-LS was either equal or better (i.e., lower) than the localization error of the P-Inv. The maximum median error difference occurred for location 37. At this location, the improvement of the VS-LS with respect to the P-Inv reached 381.8 mm. Figure 9 shows a spatial representation of the impact localization for seven locations (2, 6, 18, 20, 30, 36 and 40). For each location, the four localized impacts are indicated by a ×. Their spatial mean and median value are indicated by the symbols □ and ⟡, respectively. Looking at location 2 (in red), the VS-LS (Figure 9a) localizes three of the four impacts in the correct location while one impact is erroneously placed in location 7. Consequently, the spatial mean (red □) is closer to the actual impact location (2), while the spatial median (red ⟡), which is less influenced by outliers, coincides with the actual impact location. For the same actual impact location 2, the P-Inv method (Figure 9b) shows a localization scatter that is much more evident. Such a behavior occurs also for the other impact locations with the exception of location 20 for which the P-Inv approach works better than the VS-LS. Generally speaking, apart from location 20, the VS-LS location scattering is distributed over the direct neighbour points. Conversely, the P-Inv localization scattering is considerably wider and inconsistent. From the above discussion, it is clear that a general trend exists in the results provided by the P-Inv technique: this method is not suitable for the identification of the correct impact location. These results are in agreement with the theoretical expectations. In fact, the P-Inv provides a least-square solution of the inverse problem described in Equations (3) and (4), and, therefore, it lacks the sparsity feature that is necessary to identify point forces like impacts. This means that, in the P-Inv method, the input energy (associated to an impact) is distributed over a region on the structure, instead of pointing only to the impacted location. In addition, the location identified by P-Inv corresponds to the maximum of the estimated force indexes over the entire possible locations. It is possible that the maximum force index computed by P-Inv does not occur on the correct impact location, but on some adjacent ones. This might explain the P-Inv localization outputs for locations 30–41 (Figure 6 and Figure 7 bottom). Conversely, the VS-LS method estimates a sparse solution of the force vector and thus it is ideal for inverse problems such as point impact identification. The higher localization performance of the VS-LS is obtained thanks to the so-called “grouping effect”. This means that all of the frequency content related to a specific impact location is treated within a group. Nevertheless, there are some force scenarios that are not correctly identifiable with VS-LS (impact locations 6, 20 and 30 in Figure 7 top). The authors believe that this is mainly associated with the model-based approach.

**Figure 10:**
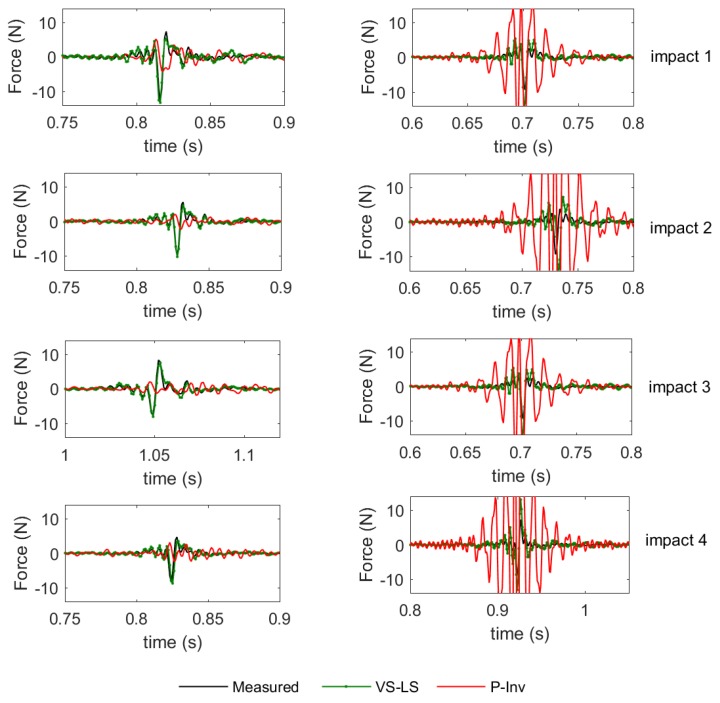
Reconstruction of the time domain forces for impact locations 7 (**left**) and 24 (**right**).

Besides the impact localization, another fundamental task of our impact identification was the reconstruction of the time history of the impact forces. As an example, the time domain results for locations 7 and 24 are presented in Figure 10. In this figure, the measured impact force is indicated by a black line while the reconstructed forces are reported in green for the VS-LS and in red for the P-Inv. For each location, the reconstructions of the four impact forces are displayed. Clearly, the VS-LS outperforms the P-Inv approach.

One of the reasons why this is happening is the fact that the reconstruction accuracy of the applied force is very dependent on the way forces have been localized. If the algorithm does not find the correct force location, the resulting time domain signal is far away from the reality. The low accuracy of the time domain reconstruction for location 7 (Figure 10 left) is mainly due to bad localization. Note that only the signal reconstructed at the identified impact location has been plotted. As P-Inv has the tendency to spread the energy over multiple locations, it is possible that certain frequencies of the identified impact location are not excited. Due to the nature of P-Inv, this excitation energy might be present in other (adjacent) impact-locations on the plate. On the other hand, as VS-LS correctly located the impact (at location 7), the reconstruction in the time domain is also more accurate. Another reason for which the reconstruction capabilities of the P-Inv are worse might be due to numerical instabilities associated with the algorithm, which could partially explain the oscillation behaviour of the P-Inv solution for location 24.

**Figure 11:**
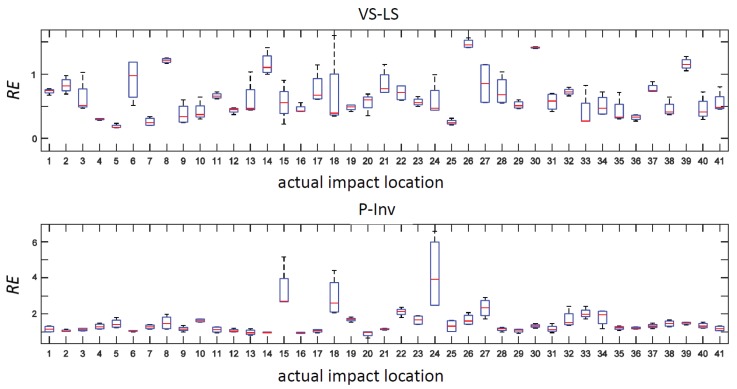
Relative error RE. Comparison between the VL-LS (**top**) and P-Inv (**bottom**) methods.

**Figure 12:**
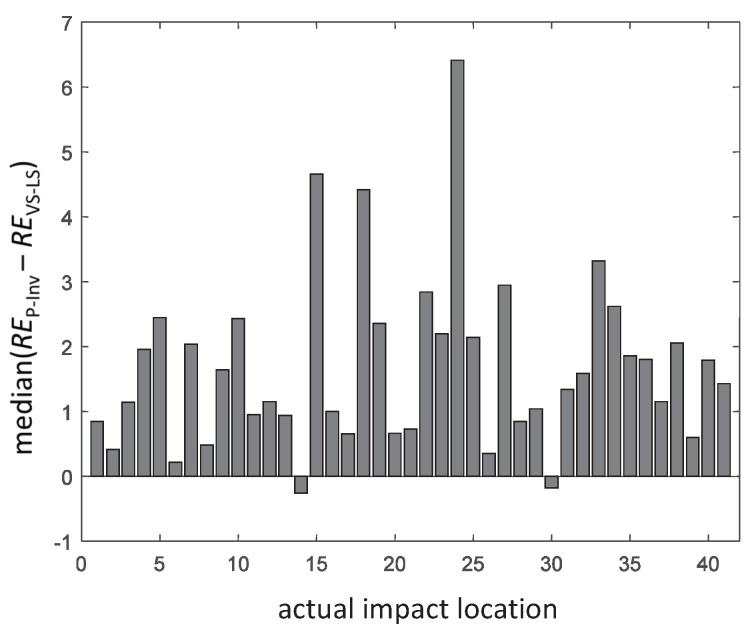
Median difference between P-Inv and VS-LS in terms of relative error RE in time domain.

To quantify the error with the time domain reconstruction, we defined the following relative error (RE) metric:

(5) $$RE=\frac{∥F-\hat{F}{∥}_{2}}{{∥F∥}_{2}},$$

where *F* and $\hat{F}$ indicate, respectively, the measured and reconstructed time history of the impact force. The box-and-whisker plot of RE is shown in Figure 11 while the median difference between RE values obtained using the P-Inv and VS-LS methods is reported in Figure 12. In 95.1% of the cases, the VS-LS provided a better time domain reconstruction of the impact forces. The highest difference between the VS-LS and the P-Inv occurred for location 24, for which the relative error of the VS-LS was 6.52 times smaller than that achieved by the P-Inv. For locations 14 and 30, the RE of the P-Inv was lower (i.e., better) than the RE of the VS-LS, although for these two locations, the P-Inv impact localization was completely unreliable (see Figure 7, Figure 8 and Figure 9).

## 5. Conclusions

In this paper, we performed quantitative impact identification on a carbon fiber reinforced stiffened composite panel with embedded FBG sensors by means of a frequency based model-dependent approach. For the analyses, we used an advanced signal processing approach which combines the fast phase correlation (FPC) algorithm for FBG spectral signal demodulation and the variable selective least square (VS-LS) method for impact localization and reconstruction. By studying 164 different impact scenarios, we showed the statistical reliability of the proposed approach, which outperforms, by far, the pseudo-inverse method conventionally used for inverse problem solving. In particular, we showed that our methodology performed better than the conventional one in 70.7% of the cases in terms of localization, and in 95.1% of the cases in terms of impact force reconstruction.

It is worth underlining that the methodology used in this work is “sensor-blind”, meaning that it is completely independent from the knowledge of the sensors’ location. Such a characteristic is particularly desirable for composites with embedded FBGs where the control on the sensor placement is somewhat limited by the manufacturing process. On the other hand, the methodology is model dependent: the better the constructed structural model, the higher the impact identification performance. In order to have a reliable structure model, in this research, we used the state-of-the-art polyreference least-square complex frequency-domain (PolyMAX) estimator.

Before concluding, it is also worth mentioning that, as all model based techniques, our impact identification technique might be affected by changes of the operating conditions caused by temperature excursions, modification of the boundary conditions, onset and propagation of damages. All of these events, in fact, produce a shift of the structural modal parameters and therefore a modification of the structure model $H^{\epsilon f}$. If these modifications are limited, the effect on the impact identification is negligible. According to the authors, temperature induced modifications should be more critical, since their effects are already visible at a low frequency. On the contrary, small damage is expected to be less relevant, since it induces relevant modifications on high order (i.e., high frequency) modes, which are generally not included in the constructed $H^{\epsilon f}$ model.

One way to take into account the influence of changing operating conditions is to retrieve the $H^{\epsilon f}$ model from a reliable finite element model of the structure under test. In such a way, different $H^{\epsilon f}$ can be obtained at different temperature conditions. Future studies will investigate the sensitivity of the methodology to slight model modifications induced by environmental changes (such as temperature modifications) and internal damage (such as delaminations and cracks).

## Figures and Tables

**Table 1:** Mechanical properties of M10/T300 carbon pre-preg.

E_11_ (GPa)	E_22_ (GPa)	$\nu_{12}=\nu_{13}$	$\nu_{21}$	$\nu_{23}$	G_11_ (GPa)	G_23_ (GPa)
125	7	0.3	0.016	0.39	3.2	2.52
